# Investigation of in vitro histone H3 glycosylation using H3 tail peptides

**DOI:** 10.1038/s41598-022-21883-0

**Published:** 2022-11-10

**Authors:** Jona Merx, Jordi C. J. Hintzen, Giordano Proietti, Hidde Elferink, Yali Wang, Miriam R. B. Porzberg, Daan Sondag, Nurgül Bilgin, Jin Park, Jasmin Mecinović, Thomas J. Boltje

**Affiliations:** 1 Institute for Molecules and Materials, Radboud University, Heyendaalseweg 135, 6525 AJ Nijmegen, The Netherlands; 2 Department of Physics, Chemistry and Pharmacy, University of Southern Denmark, Campusvej 55, 5230 Odense, Denmark; 3 Department of Blood Transfusion, China-Japan Union Hospital, Jilin University, 126 Xiantai Street, Changchun, 130033 China; 4 College of Natural & Applied Science, University of Guam, Mangilao, Guam, 96913 USA

**Keywords:** Amino sugars, Monosaccharides, Glycobiology, Epigenetics

## Abstract

Posttranslational modifications (PTMs) on histone tails regulate eukaryotic gene expression by impacting the chromatin structure and by modulating interactions with other cellular proteins. One such PTM has been identified as serine and threonine glycosylation, the introduction of the ß-*N*-acetylglucosamine (GlcNAc) moiety on histone H3 tail at position Ser10 and Thr32. The addition of the ß-*O*-GlcNAc moiety on serine or threonine residues is facilitated by the *O*-GlcNAc transferase (OGT), and can be removed by the action of *O*-GlcNAcase (OGA). Conflicting reports on histone tail GlcNAc modification in vivo prompted us to investigate whether synthetic histone H3 tail peptides in conjunction with other PTMs are substrates for OGT and OGA in vitro. Our enzymatic assays with recombinantly expressed human OGT revealed that the unmodified and PTM-modified histone H3 tails are not substrates for OGT at both sites, Ser10 and Thr32. In addition, full length histone H3 was not a substrate for OGT. Conversely, our work demonstrates that synthetic peptides containing the GlcNAc functionality at Ser10 are substrates for recombinantly expressed human OGA, yielding deglycosylated histone H3 peptides. We also show that the catalytic domains of human histone lysine methyltransferases G9a, GLP and SETD7 and histone lysine acetyltransferases PCAF and GCN5 do somewhat tolerate glycosylated H3Ser10 close to lysine residues that undergo methylation and acetylation reactions, respectively. Overall, this work indicates that GlcNAcylation of histone H3 tail peptide in the presence of OGT does not occur in vitro.

## Introduction

**Figure 1:**
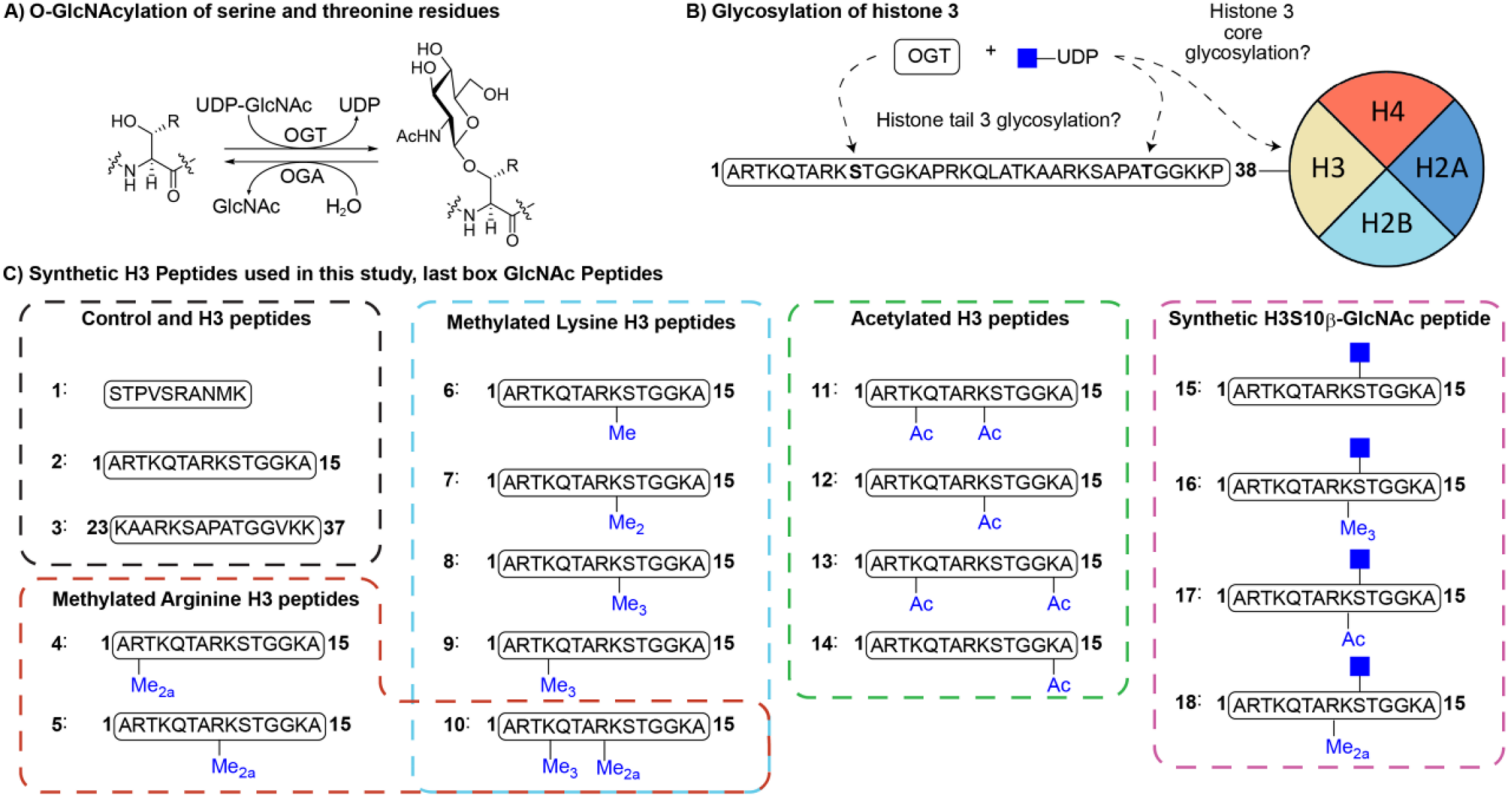
(**A**) OGT catalyzes the transfer of UDP-GlcNAc to serine/threonine residues. OGA hydrolyses the GlcNAc-possessing serine/threonine residues resulting in native serine (R = H)/threonine (R = CH_3_), (**B**) Identified sites of O-GlcNAc modification of histone H3, (**C**) Histone peptides and controls used in this study. Me = methyl, Me_2_ = dimethyl, Me_2a_ = asymmetric dimethyl, Me_3_ = trimethyl, Ac = acetyl.

DNA is spooled around histone proteins that comprise the nucleosome, the basic unit of eukaryotic chromatin^1^. The availability of DNA for gene transcription is regulated by posttranslational modifications (PTMs) on unstructured histone tails that protrude from the nucleosomal core histones H2A, H2B, H3 and H4^2^. Phosphorylation of serine and threonine residues has been demonstrated to occur on histone tails and many other nuclear proteins^3^. Many of the serine/threonine phosphorylation sites present in nuclear proteins can also be modified by the addition of β-*O*-GlcNAc residues^4^. The addition of β-*O*-GlcNAc is catalyzed by *O*-GlcNAc transferase (OGT) utilizing UDP-GlcNAc as the sugar donor, and is removed by *O*-GlcNAcase (OGA) (Fig. 1A). The competition between phosphorylation and glycosylation for serine/threonine sites leads to a crosstalk between these two PTMs^5^. For example, *O*-GlcNAcylation decreases phosphorylation of microtubule-associated TAU protein in vivo, further demonstrated by reciprocal regulation of phosphorylation by *O*-GlcNAcylation in vitro^6,7^. There are indications that histones can also be glycosylated by OGT, either at sites in the core of histone protein or its corresponding histone tail (Fig. 1B)^8–11^. The presence of β-*O*-GlcNAc residue on Ser10 in the H3 tail was demonstrated by staining with β-*O*-GlcNAc binding antibodies, mass spectrometry and bioorthogonal chemistry^10^.

The presence of a β-*O*-GlcNAc moiety on histone H3 tail might present a novel mechanism that may control transcription directly or indirectly by influencing PTMs taking place at the same or neighboring sites. As histone tails are flexible and contain a relatively modest recognition domain, showing activity for the enzymes that introduce PTMs (known as ‘writers’) or remove PTMs (known as ‘erasers’) on small peptide fragments, the core protein is of little influence in most cases and can be omitted in enzymatic assays. For example, we have shown that histone lysine methylation and acetylation can be studied on small histone tail peptides in vitro, exploring the tolerance of unnatural lysine analogs by the catalytic domains of lysine methyltransferases (KMTs) and acetyltransferases (KATs)^12–18^. Furthermore microarrays of histone tail peptide fragments are commonly used to discover interactions and interrogate with a host of histone readers^19,20^.

Studying the PTMs of histone tails can be performed in vitro on synthetic histone tail peptides for most types of PTMs^21,22^. Intrigued by the reports of histone tail glycosylation we set out to investigate whether H3 tail peptide fragments bearing Ser10 and Thr32 can indeed be modified with a β-*O*-GlcNAc residue by OGT and whether OGA would be able to remove such PTM. To this end, we prepared synthetic unmodified and glycosylated H3 peptides and tested the ability of recombinantly expressed human OGT and OGA to install and remove the β-*O*-GlcNAc moiety, respectively. We found that the H3 tail peptide fragments or full length histone H3 used in this study do not appear to be substrates for the enzymatic glycosylation by OGT, even though OGT efficiently glycosylated a smaller non-histone derived control peptide and the full length histone H2A and H2B. The influence of other naturally occurring PTMs such as lysine mono-, di- and trimethylation as well as lysine acetylation and asymmetric arginine dimethylation were also investigated but did not result in *O*-GlcNAcylation of the histone peptides. Conversely, OGA was able to catalyze the removal of the β-*O*-GlcNAc residue of synthetic H3 glycopeptides indicating that the removal is at least possible. These results suggest that OGT either requires an intact nucleosome or the presence of other nuclear proteins. Another possibility yet is that histone H3 tails are not glycosylated at all, as a study sought to validate the earlier findings of histone glycosylation however ultimately concluded that histone tails are not glycosylated in vivo in contrast to the positive controls used in their rigorous study^23^.

## Results and discussion

To investigate whether histone H3 tails are substrates for OGT in vitro and establish its dependence/crosstalk with other PTMs such as lysine methylation, lysine acetylation and arginine methylation, we set out to synthesize a panel of synthetic H3 tail peptides (2–18, Fig. 1C). Unmodified peptides 1–3 were prepared to establish the enzymatic activity of human OGT towards a positive control (1)^24^ and two H3 fragments (2 and 3, see Fig. 1C). Synthetic methylated lysine and arginine H3 peptides 4–10 were also used to this end. To probe the influence of lysine acetylation on the activity of OGT, acetylated H3 peptides 11–14 were prepared. Furthermore, the expected product 15 of OGT-catalyzed glycosylation of 2 was chemically prepared as a reference standard, and to investigate the ability of OGA to catalyze deglycosylation of 15. Finally, chemically modified peptides bearing a Ser10GlcNAc in combination with methylation or acetylation marks of neighboring Lys9 (16–17) or asymmetric dimethylated Arg8 (18) were also investigated as OGA substrates.

**Figure 2:**
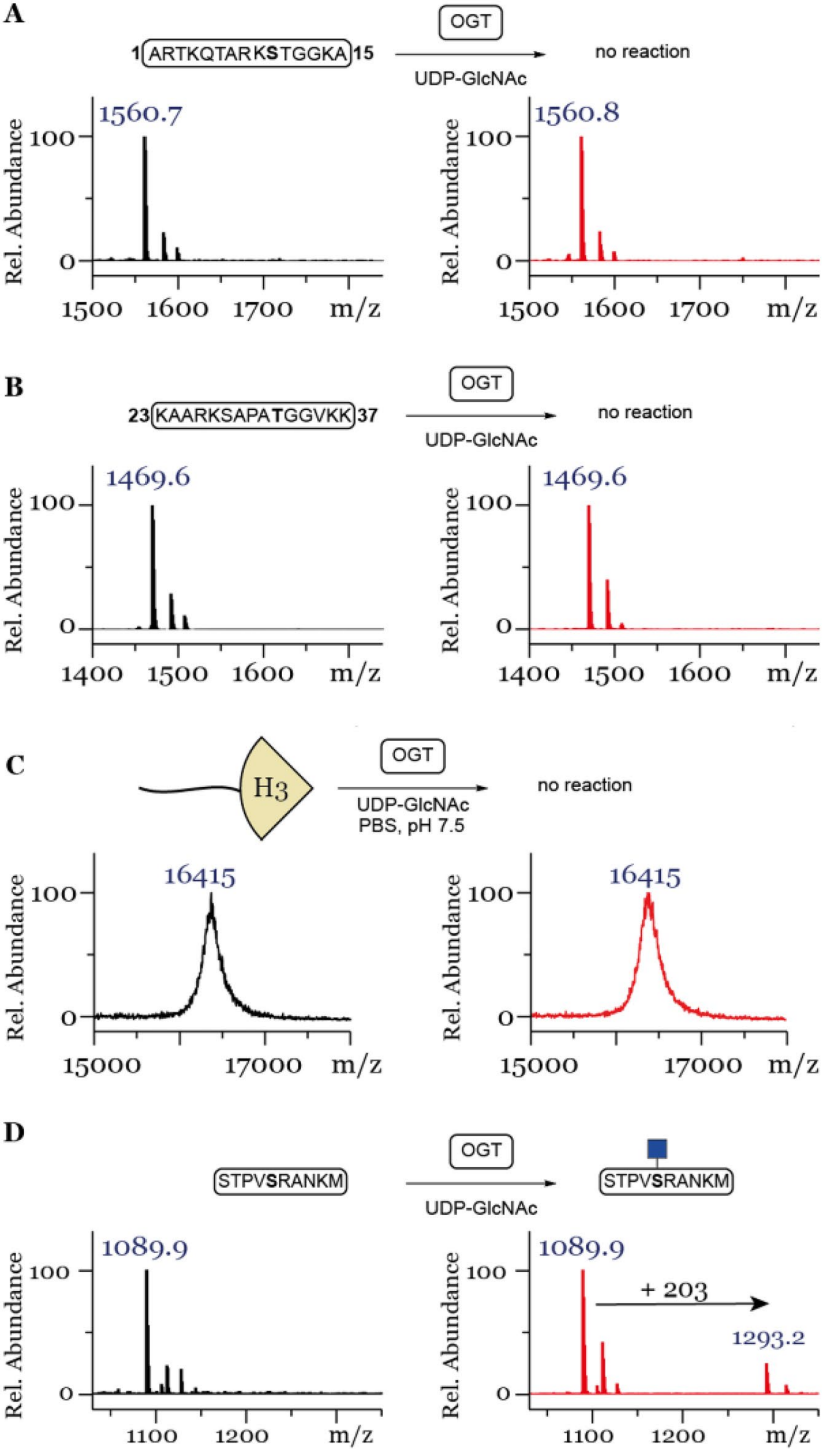
MALDI-TOF MS data showing that histone peptides (**A**) H3(1–15), (**B**) H3(23–37) and (**C**) full length histone H3 are not glycosylated by OGT, (**D**) while the control peptide shows glycosylation under the same conditions. Red spectra show 1 h reactions at 37 °C with 25 µM peptide/protein, 5 µM OGT, 50 µM UDP-GlcNAc and black spectra show the no-enzyme controls.

To investigate the potential OGT-catalyzed glycosylation of synthetic H3 peptides we turned to MALDI-TOF MS enzymatic assays, a commonly used methodology for examining PTMs on histone peptides^12–15,17^. After the enzymatic reaction is quenched, the peptides are analyzed by MALDI-TOF mass spectrometry, where a mass change corresponds to either introduction or removal of PTM(s). To determine whether this technique is appropriate for monitoring the glycosylation of synthetic H3 tail peptides, we first analyzed an equimolar mixture of peptides 2 and 15 with MALDI-TOF in the enzymatic assay buffer. It is well established that glycosylated peptides yield a lower signal in the mass spectrum compared to their non-glycosylated counterparts^25^. Indeed, the glycosylated peptide 15 displayed a five-fold reduction in signal compared to the unglycosylated counterpart 2 (Fig. S10). Nevertheless, the signal to noise ratio was more than adequate to evaluate the glycosylation of H3 tail peptides albeit in a non-quantitative manner.

**Figure 3:**
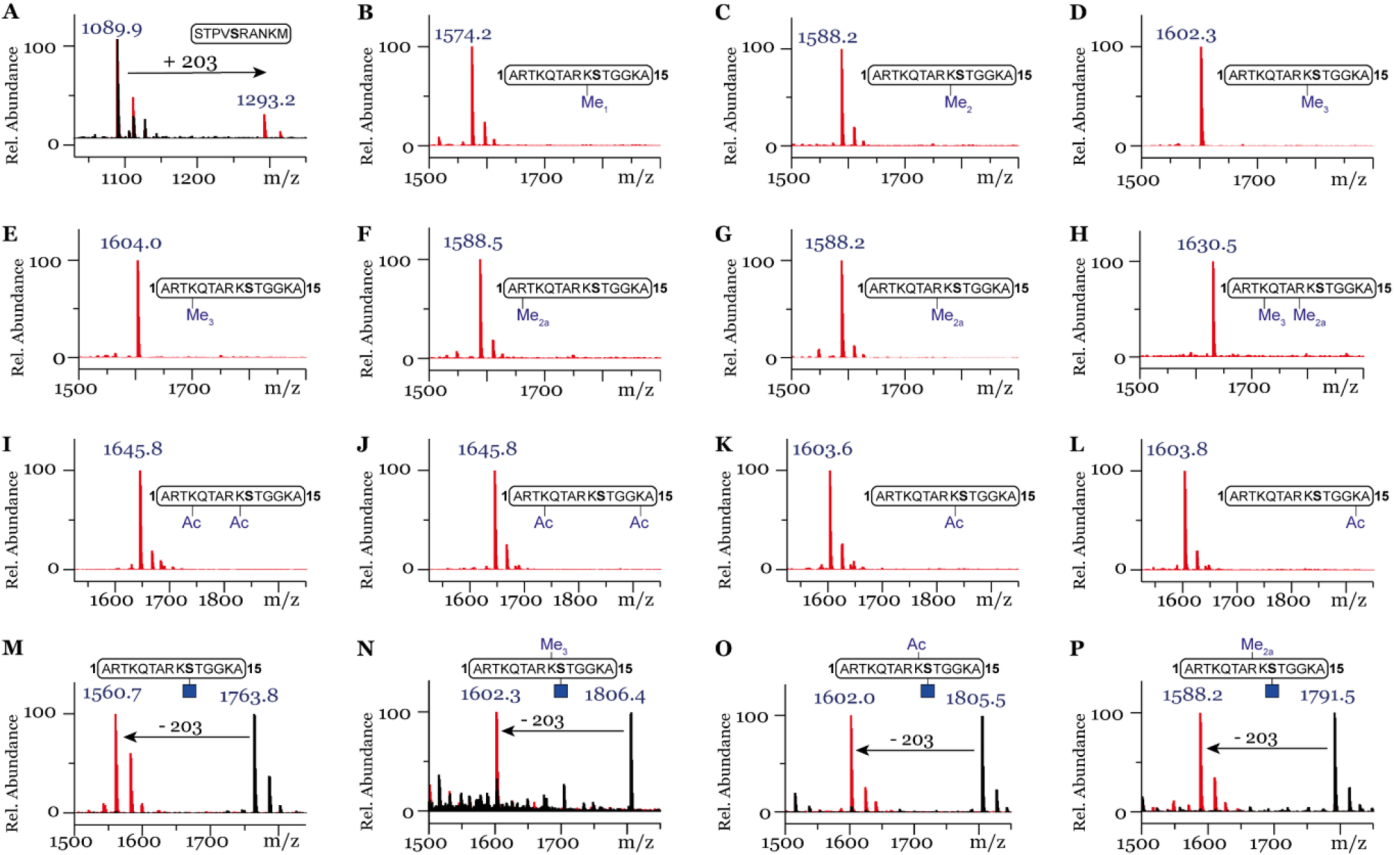
MALDI-TOF MS data showing glycosylation in the presence of OGT with (**A**) Control peptide. No glycosylation observed with peptides: (**B**) H3K9Me, (**C**) H3K9Me2, (**D**) H3K9Me3, (**E**) H3K4Me3, (**F**) H3R2Me2a, (**G**) H3R8Me2a, (**H**) H3K4Me3R8Me2a, (**I**) H3K4AcK9Ac, (**J**) H3K4AcK14Ac, (**K**) H3K9Ac, (**L**) H3K14Ac. OGA efficiently hydrolyses (**M**) H3S10GlcNAc, (**N**) H3K9Me3S10GlcNAc, (**O**) H3K9AcS10GlcNAc, (**P**) H3R8Me2aS10GlcNAc. Red spectra show 1 h reactions at 37 °C with 5 µM OGT, 50 µM UDP-GlcNAc (**A**–**L**) or with 1 µM OGA (**M**–**P**) and black spectra the no-enzyme controls.

Having established that we can indeed detect glycosylation of the reference peptides with MALDI-TOF MS, we investigated the ability of OGT to catalyze glycosylation of the unmodified H3 peptide. First, we validated the activity of human OGT by incubating a known control peptide 1^24^ with OGT showing significant enzymatic glycosylation after 1 h (Fig. S11), however, complete conversion was never obtained even upon 3 h incubation, as indicated by a lack of the mass shift of + 203 Da (Fig. 2D). The use of other buffers, commonly used for OGT promoted *O*-GlcNAcylation reactions did not result in glycosylation of the unmodified histone H3 tail peptide **2** (Fig. S12), therefore for all subsequent reactions PBS (pH 7.4) was used. As prolonged incubation time did not increase glycosylation on the positive control all the subsequent enzymatic reactions were quenched and analyzed after 1 h only. Next, peptides 2 (Fig. 2A) and 3 (Fig. 2B) were incubated under the standard conditions (25 µM peptide, 1 µM OGT, 30 µM UDP-GlcNAc, Fig. S13) or the optimized conditions using a higher concentration of enzyme and co-substrate (25 µM peptide, 5 µM OGT, 50 µM UDP-GlcNAc, Fig. 2). MALDI-TOF MS data showed that neither peptide showed a mass increase corresponding to GlcNAcylation, indicating that Ser10 and Thr32 were not glycosylated, respectively. To investigate whether a full length histone context is necessary to observe glycosylation, histones H2A, H2B, H3 and H4 were incubated under the optimized conditions (25 µM histone, 5 µM OGT, 50 µM UDP-GlcNAc). Histones H2A and H2B showed a mass increase corresponding to the addition of GlcNAc, whereby H2A was amenable to modification with two GlcNAc residues (Fig. S14A,B). However, full length histone H3 subjected to the same conditions did not show a sign of OGT- catalyzed *O*-GlcNAcylation (Fig. 2C). Similarly, no OGT catalysed glycosylation was observed in the case of histone H4 (Fig. S14C).

As histone tails are subjected to numerous PTMs in vivo, we next investigated the possibility that H3 PTMs are required for H3 glycosylation with a focus on the Ser10 position. First, we investigated the effect of methylation of neighboring lysine H3K9 and arginine H3R8 residues, while also considering the methylation of H3K4. To this end the H3 peptide containing different methylation states of H3K9 (6, 7, 8) and trimethylated H3K4 (9) were synthesized. Furthermore, H3 peptides containing asymmetrically dimethylated Arg2Me2a (4), and asymmetrically methylated Arg8Me2a (5), the latter also combined with trimethylated K4Me3 (10) were synthesized by SPPS and further purified by preparative HPLC. The enzymatic conversions by OGT for this set of H3 peptides were evaluated under optimized conditions (5 µM OGT, 50 µM UDP-GlcNAc).

Among the tested methylated histone peptides (4–10) bearing different methylation marks, none were observed to be glycosylated in the presence of higher concentration of OGT (Fig. 3B–H) whereas the control did show conversion (Fig. 3A). Another common modification of lysine residues on H3 tail peptides is acetylation, which leads to a decrease in the charge state (neutral) under physiological conditions and was hence also investigated^26^. To this end, synthetic peptides 11–14 were incubated with OGT under high concentration assay conditions. Neither mono-acetylated neighboring K9 (12) or K14 (14) residues nor diacetylated H3 peptides (11 and 13) yielded glycosylated peptides (Fig. 3I–L).

While unable to detect glycosylation by OGT of histone peptides of H3 in vitro, we continued the investigation by testing the synthetic glycosylated peptides 15–18 with OGA, the enzyme which is responsible for the removal of the β-*O*-GlcNAc moiety from peptide/protein substrates. Interestingly, peptides 15–18 were found to be good substrates for OGA as incubation with OGA resulted in efficient enzymatic hydrolysis of GlcNAc, yielding deglycosylated H3 peptides (Fig. 3M–P).

**Figure 4:**
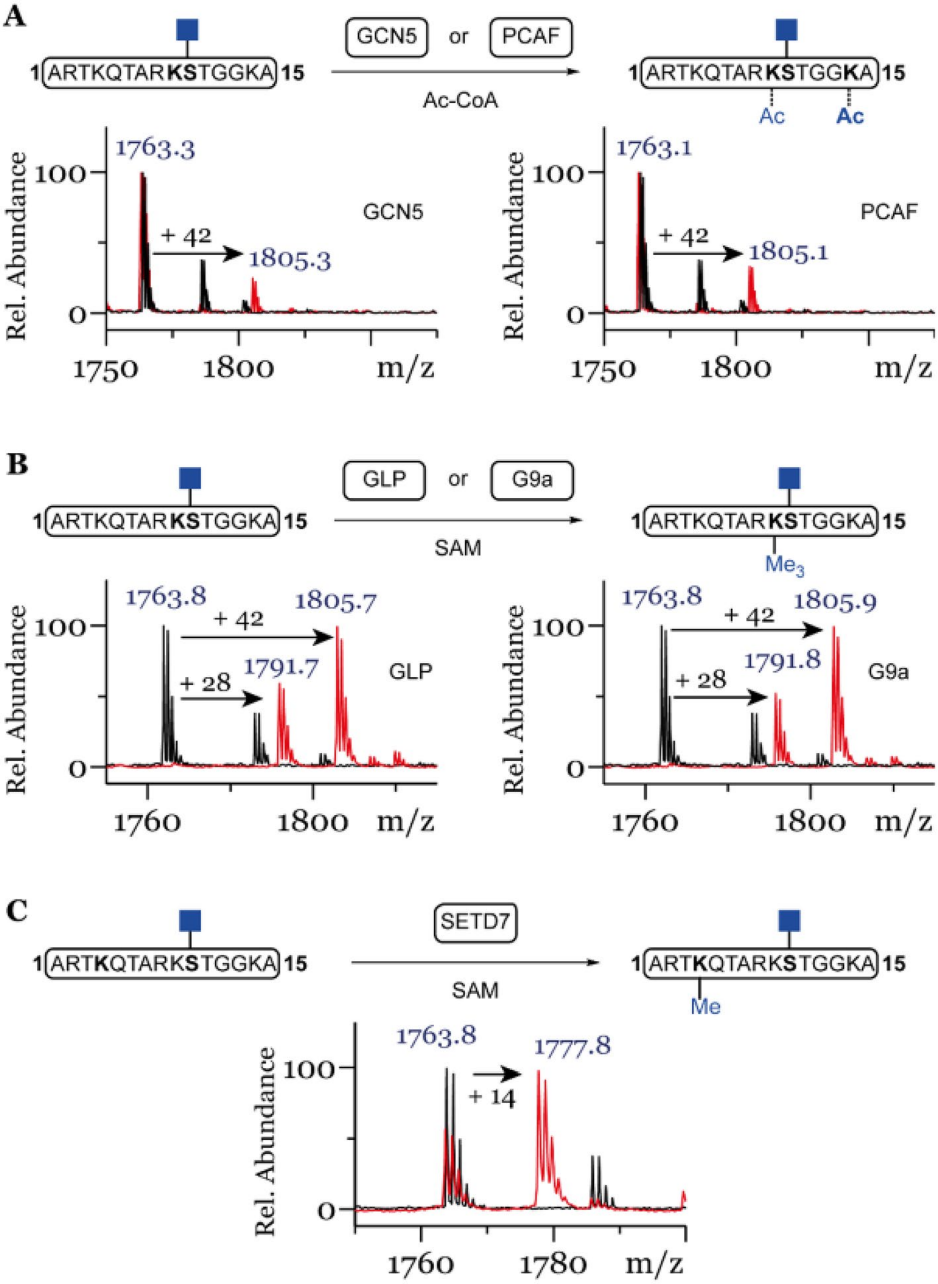
(**A**) Mono-acetylation of H3S10ßGlcNac by lysine acetyltransferases GCN5 or PCAF, (**B**) Di- and tri-methylation of H3S10ßGlcNac by lysine methyltransferases GLP or G9a, (**C**) Mono-methylation of H3S10ßGlcNac by lysine methyltransferase SETD7. Red spectra show 3 h reactions at 37 °C with KATs (2 uM), histone peptide (100 µM) and Ac-CoA (300 µM) (**A**) or 1 h incubations with KMTs (2 µM), SAM (500 µM for GLP/G9a and 200 µM for SETD7) (**B**,**C**) of histone peptide 15 (100 µM) and black spectra the no-enzyme controls.

To investigate whether Ser10 glycosylation has an effect on introduction of neighboring PTMs, we tested the glycosylated H3S10 peptide with the catalytic domain of histone lysine acetyltransferases PCAF (lysine acetyltransferase 2B) and GCN5 (lysine acetyltransferase 2A). Both enzymes are able to acetylate unmodified peptide preferentially at K14 and K9^17,27,28^. After 3 h incubation, low degree of mono-acetylation of glycosylated peptide 15 was observed for GCN5 and to a slightly greater extend for PCAF (Fig. 4A). Furthermore, we tested the glycosylated peptide 15 with the catalytic domain of histone lysine methyltransferases SETD7, GLP and G9a. Surprisingly the glycosylated peptide 15 was amenable to mono-methylation of K4 by SETD7 (Fig. 4C) and efficient di- and tri-methylation of K9 by GLP and G9a (Fig. 4B), in line with the observed activity of these enzymes for unmodified H3 peptide 2^13^.

The inability of OGT to glycosylate histone tail H3 peptides or full length histone H3 in vitro, while amenable to efficient hydrolysis of glycosylated H3 peptides by action of OGA, contributes to the discussion whether histones are indeed glycosylated^23^. As full length histone H3 was not amenable to glycosylation, other proteogenic factors serving as template may be required for activity, such as TET2^29^. Approaches to characterize the extent of the GlcNAc modification in the proteome fail to identify histone H3 in several untargeted methodologies^30–33^. In contrast, when OGA resistant metabolic chemical reporters (MCRs) are used histone H3 was identified in one case^34^, however, it resulted in no identification using a similar method^35^. Recent realizations that acetylated MCRs can react non-enzymatically with cysteine residues, complicates the interpretive power of these tools^36,37^. Combined with our in vitro results, the validity of histone H3 tail *O*-GlcNAcylation, especially H3S10, is tenuous.

## Conclusion

Our enzymatic assays demonstrate that human OGT is unable to catalyze glycosylation of unmodified histone H3 peptides (residues 1–15 and 23–37) in vitro, including H3 peptides bearing lysine methylation (K4Me3, K9Me1-3) arginine methylation (R2Me2a, R8Me2a), combined lysine and arginine methylation (H3K4Me3R8Me2a) or lysine acetylation (K4Ac, K9Ac, K4AcK9Ac, K4AcK14Ac) marks on neighboring or more distant lysine and arginine residues. Full length histone H3 was also not amenable to OGT-catalyzed glycosylation, whereas full length histone H2A and H2B were glycosylated under the same conditions. Conversely, chemically synthesized *O*-GlcNAc H3 peptides showed that human OGA has a capacity to efficiently remove the GlcNAc group from the Ser10GlcNAc residue at H3. Furthermore, the modification of H3 peptides bearing GlcNAc at Ser10 is amenable to further modification by the catalytic domains of histone lysine methyltransferases SETD7, G9a and GLP, and to a lesser extent modification by histone lysine acetyltransferases GCN5 and PCAF. These results indicate that human OGA tolerates common PTMs placed close to the glycosylated serine residue H3S10 that undergoes deglycosylation reaction. The inability of OGT to catalyze glycosylation of H3 peptides in vitro indicates that either histone H3 is not glycosylated in vivo supporting the work of Gagnon et al*.*^23^ or that additional factors/proteins, such as chaperones, are required for efficient glycosylation of histone proteins in vivo. The results described here contribute to the ongoing discussion whether the glycosylation of histone tail of H3 is misidentified.

## Experimental

### Synthesis of building blocks and peptides

Fmoc-Ser-OAllyl^38^: To a cooled (0 °C) solution of Fmoc-serine-OH (3.0 g, 9.17 mmol) in dry DMF (40 ml), trimethylamine (2.5 mL, 18.3 mmol) and allyl bromide (1.6 mL, 18.3 mmol) were added dropwise. The reaction stirred for 16 h at rt before diluting with EtOAc (100 mL). The reaction was washed with water (4 × 100 mL), brine (100 mL), dried (Na_2_SO_4_), filtered and concentrated in vacuo. The mixture was purified by column chromatography (50% EtOAc in Heptane) to afford the product as a white solid in 88% yield (2.97 g, 8.08 mmol). *Rf* 0.4 (1:1 EtOAc:Heptane); ^1^H NMR (400 MHz, DMSO-d6) δ 7.89 (d, *J* = 7.5 Hz, 2H), 7.73 (dd, *J* = 7.5, 3.6 Hz, 2H), 7.61 (d, *J* = 8.0 Hz, 1H), 7.47–7.38 (m, 2H), 7.33 (tt, *J* = 7.4, 1.4 Hz, 2H), 5.89 (ddt, *J* = 17.2, 10.5, 5.2 Hz, 1H), 5.32 (dq, *J* = 17.3, 1.7 Hz, 1H), 5.19 (dq, *J* = 10.5, 1.5 Hz, 1H), 5.05–4.91 (m, 1H), 4.59 (dt, *J* = 5.2, 1.6 Hz, 2H), 4.37–4.12 (m, 4H), 3.69 (t, *J* = 5.4 Hz, 2H); ^13^C NMR (101 MHz, DMSO-d6) δ 170.5, 156.1, 143.81, 143.78, 140.7, 132.8, 127.7, 127.1, 125.3, 125.2, 120.1, 117.5, 65.8, 64.8, 61.2, 56.8, 46.6, 30.7.

*ß*-GlcNAc-OAc_3_: To a solution of (3*R*,4*R*,5*S*,6*R*)-6-(acetoxymethyl)-3-aminotetrahydro-2H-pyran-2,4,5-triyl triacetate hydrochloride (4.85 g, 12.6 mmol) in pyridine (30 mL) was added Ac_2_O (1.31 mL, 13.9 mmol). After stirring for 16 h the solution was concentrated in vacuo, dissolved in EtOAc and washed with 1 M HCl. The organic layer was dried over MgSO_4_, filtered and concentrated in vacuo to obtain a white solid in 87% yield (4.26 g, 10.95 mmol). ^1^H NMR (500 MHz, CDCl_3_) δ 6.12 (d, *J* = 9.6 Hz, 1H), 5.68 (d, *J* = 8.8 Hz, 1H), 5.18 (dd, *J* = 10.6, 9.4 Hz, 1H), 5.07 (t, *J* = 9.7 Hz, 1H), 4.34–4.16 (m, 2H), 4.09 (dd, *J* = 12.5, 2.3 Hz, 1H), 3.83 (ddd, *J* = 9.9, 4.8, 2.3 Hz, 1H), 2.07 (s, 3H), 2.04 (s, 3H), 2.01 (s, 3H), 2.00 (s, 3H), 1.89 (s, 3H); ^13^C NMR (126 MHz, CDCl_3_) δ 171.3, 170.8, 170.3, 169.6, 169.4, 92.5, 72.8, 72.7, 68.1, 61.8, 52.9, 23.2, 20.9, 20.8, 20.7, 20.6.

Fmoc-Ser(*ß*-GlcNAc-OAc_3_)-OAllyl^39^: Fmoc-Ser-OAllyl (2.5 g, 6.8 mmol) was added to a dry solution of *ß*-GlcNAc-OAc_3_ (2.0 g, 5.2 mmol) and Sc(OTf)_3_ (260 mg, 0.52 mmol) in DCE (30 mL). The mixture was refluxed for 72 h. The mixture was diluted with DCM and washed with water, followed by extraction of the aqueous layer with DCM. The combined organic layers were dried over MgSO_4_, filtered, concentrated in vacuo and purified by column chromatography (30 to 70% EtOAc in Heptane) to yield a crystalline white solid in 58% yield (2.1 g, 5.2 mmol). *Rf* = 0.3 (3:1 EtOAc:Hept.); ^1^H NMR (400 MHz, CDCl_3_) δ 7.78 (ddd, *J* = 7.6, 2.7, 1.8 Hz, 2H), 7.68–7.62 (m, 2H), 7.45–7.38 (m, 2H), 7.33 (tt, *J* = 7.4, 1.1 Hz, 2H), 5.89 (ddt, *J* = 16.4, 10.8, 5.6 Hz, 1H), 5.78 (d, *J* = 8.3 Hz, 1H), 5.50 (d, *J* = 8.5 Hz, 1H), 5.37–5.30 (m, 1H), 5.28–5.21 (m, 2H), 5.04 (t, *J* = 9.6 Hz, 1H), 4.74–4.61 (m, 3H), 4.55–4.41 (m, 3H), 4.30–4.19 (m, 3H), 4.17–4.05 (m, 1H), 3.86 (dd, *J* = 10.5, 3.3 Hz, 1H), 3.80 (dt, *J* = 10.6, 8.4 Hz, 1H), 3.67 (ddd, *J* = 10.0, 4.8, 2.5 Hz, 1H), 2.07 (s, 3H), 2.04 (d, *J* = 1.9 Hz, 6H), 1.85 (s, 3H); ^13^C NMR (126 MHz, CDCl_3_) δ 170.0, 170.8, 170.7, 169.5, 169.4, 156.2, 144.0, 143.8, 141.5, 141.4, 131.6, 127.9, 127.3, 127.2, 125.3, 125.3, 120.2, 120.1, 118.8, 101.0, 72.3, 72.1, 69.1, 68.6, 67.0, 66.5, 62.2, 54.7, 54.4, 47.4, 23.3, 20.9, 20.8, 20.8.

Fmoc-Ser(ßGlcNAc-OAc_3_)-OH^40^: To a solution of Fmoc-Ser(ß-GlcNAc-OAc3)-OAllyl (2.0 g, 2.9 mmol) in DCM (20 mL) was added Pd(PPh_3_)_4_ (33 mg, 0.029 mmol). The mixture was purged with Argon. Morpholine (0.50 mL, 5.7 mmol) was added over a period of 5 min. The solution was stirred for 40 min after which it was diluted with DCM and washed with 0.1 M KHSO_4_. The organic layer was dried over MgSO_4_, filtered, concentrated in vacuo and purified by column chromatography (0 to 5% MeOH in DCM + 1% AcOH). After lyophilization a fluffy white solid was obtained in 93% yield (1.8 g, 2.7 mmol). *Rf* = 0.6 (10% MeOH in DCM + 1% AcOH); ^1^H NMR (500 MHz, MeOD-d4) δ 7.80 (d, *J* = 7.6 Hz, 2H), 7.68 (t, *J* = 6.3 Hz, 2H), 7.45–7.37 (m, 2H), 7.32 (tdd, *J* = 7.5, 2.7, 1.1 Hz, 2H), 5.23 (dd, *J* = 10.5, 9.2 Hz, 1H), 4.97 (dd, *J* = 10.0, 9.3 Hz, 1H), 4.72 (d, *J* = 8.4 Hz, 1H), 4.42 (dd, *J* = 10.5, 6.9 Hz, 1H), 4.39–4.20 (m, 4H), 4.16–4.08 (m, 2H), 3.97–3.89 (m, 1H), 3.87–3.72 (m, 2H), 2.04–2.02 (m, 3H), 2.00 (s, 3H), 1.97 (s, 3H), 1.86 (s, 3H); ^13^C NMR (126 MHz, MeOD-d4) δ 173.7, 172.4, 171.8, 171.3, 158.4, 145.3, 145.2, 142.6, 128.8, 128.24, 128.22, 126.3, 126.2, 121.1, 101.9, 74.0, 73.0, 70.1, 67.7, 63.2, 55.4, 22.9, 20.6, 20.6, 20.5.

All peptides were prepared via standard Fmoc solid-phase peptide synthesis (SPPS) with either commercially available Fmoc-Lys(Ac)-OH, Fmoc-protected methylated lysines and arginines or Fmoc-Ser(β-O-GlcNAc)-OH (4). Deprotection of the O-Acetyl protection groups of peptides prepared from Fmoc-Ser(β-O-GlcNAc)-OH was performed with 15% hydrazine in MeOH overnight before the final cleavage step. Synthetic histone H3 peptides were purified by preparative HPLC, and their purity and identity verified by analytical HPLC and mass spectrometry (Figs. S5–9).

### Recombinant protein expression

OGA and OGT were expressed and purified as previously described^41^. pET28a containing either OGT or OGA was transformed into BL21(DE3) *E. coli*. Cultures were grown at 37 °C in TB medium for OGT and LB for OGA containing a final concentration of kanamycin of 50 µg/mL. Cells were grown to a OD_600_ of 1.5–1.7. The culture was cooled to 16 °C, expression was induced with IPTG (0.2 mM final concentration) and shaken overnight. After harvesting of the cells and sonication after addition of cOmplete™ Protease Inhibitor Cocktail, the insoluble debris was removed via centrifugation. The clear lysate was purified with a Ni–NTA affinity chromatography column with fractional collection. The pure fraction containing the protein were concentrated using Amicon Ultra Centrifugal Filter Unit (30 KD cutoff), flash frozen and stored at −80 °C. Typical yield of the expression 1–2 mg/L culture. See SI Fig. S15 for a coommasie staining of the expressed OGT.

Catalytic domains of human KMTs (G9a, GLP, SETD7) were expressed and purified as previously described^42–44^. The methyl transferase plasmid (SETD7 residues 1–366, GLP residues 951–1235, G9a residues 913–1193) transformed into Escherichia coli Rosetta BL21 (DE3)pLysS cells. Cultures were grown at 37 °C in LB media containing kanamycin and chloramphenicol. Cells were grown to an OD_600_ of 0.5–0.6, at which point they induced by IPTG (1.0 mM) and they were transferred to a temperature of 16 °C overnight. After letting the cells grow at this temperature, they were then harvested and lysed by sonication. The lysate was centrifuged at high-speed to remove unbroken cells. The supernatant was then centrifuged to further clean the lysate. Purification of the N-terminally his6-tagged KMTs was performed using Ni–NTA affinity chromatography column, which was prewashed with lysis buffer. Target KMTs enzyme was then eluted using a linear gradient concentration of imidazole. The elute was then concentrated with centrifugal concentrators (Millipore). All KMTs enzymes were further purified by gel filtration on a Superdex 75 column (GE Healthcare) on an AKTA system. Purified proteins were concentrated employing Amicon Ultra Centrifugal Filter Units (Millipore) with suitable molecular weight cutoffs.

Plasmid carrying recombinant His-tagged human GCN5 catalytic domain (residues 497–662 in pET28a-LIC vector) was purchased from Addgene (25,482). The protein was expressed and purified as previously described^45^. Briefly, *E. coli* BL21(DE3) cells enriched with GCN5 WT plasmid were cultured in TB growth media supplemented with 50 µg/mL kanamycin at 37 °C to an OD_600_ of 0.6, upon which expression was induced by addition of IPTG (0.5 mM final) and followed by incubation at 16 °C overnight. Harvested cells were pelleted and re-suspended into 50 mM Na_2_HPO_4_ pH 7.5, 500 mM NaCl, 5% glycerol, 1 mM β-ME lysis buffer in presence of protease inhibitor cocktail (Roche, Basel, Switzerland) and lysate by sonication. The supernatant was incubated with Ni–NTA beads for 2 h at 4 °C. The beads were loaded on a gravity flow column and washed with 20 mM HEPES–NaOH pH 7.5, 500 mM NaCl, 50 mM imidazole, 5% glycerol, 1 mM β-ME. Subsequently, the protein was eluted with 20 mM HEPES–NaOH pH 7.5, 500 mM NaCl, 250 mM imidazole, 5% glycerol, 1 mM β-ME and the buffer was exchanged to 20 mM HEPES–NaOH pH 7.5, 150 mM NaCl, 1 mM βME by concentration with a 10 kDa spinfilter device (AMICON, 10 MWCO). Purity of the eluted protein was assessed with SDS-page, and GCN5 was separated in aliquots, rapidly flash-frozen and stored at −80 °C.

Plasmid carrying recombinant SNAP-His-tagged human PCAF catalytic domain (residues 498–658 in pET16b vector) was provided by Professor Milan Mrksich (Northwestern University). *E. coli* BL21(DE3) cells enriched with PCAF plasmid, were cultured in 2xYT growth media supplemented with 100 µg/mL carbenicillin at 37 °C, to an OD600 of 0.6, upon which expression was induced by addition of IPTG (0.5 mM final) and followed by incubation at 16 °C overnight. Harvested cells were pelleted and re-suspended into 50 mM Tris pH 8.5, 200 mM NaCl, 5 mM β-ME lysis buffer in presence of protease inhibitor cocktail (Roche) and lysate by sonication. The supernatant was incubated with Ni–NTA beads for 2 h at 4 °C. The beads were loaded on a gravity flow column and washed with 50 mM Tris pH 8.5, 200 mM NaCl, 50 mM imidazole, 5 mM β-ME. Subsequently, the protein was eluted with 50 mM Tris pH 8.5, 200 mM NaCl, 300 mM imidazole, 5 mM β-ME and concentrated using a 30 kDa spinfilter device (AMICON, 30 MWCO). The protein was furtherly purified by size-exclusion chromatography (SEC) using the AKTA system, employing a Superdex 75 column equilibrated with 50 mM Tris pH 8.0, 200 mM NaCl, 1 mM DTT at 0.5 mL/min flow speed. The purity of the eluted protein was assessed with SDS-page. Pure fractions were pooled, rapidly flash-frozen and stored at −80 °C.

Full length histone H2A, H2B and H4 (*X. laevis*) were prepared as described previously^46^, while human histone H3 was acquired commercially (Sigma-Aldrich, SRP0177). The lyophilized powder was dissolved at a concentration of 1 mM and used in the full-length histone MALDI-TOF MS assays.

### MALDI-TOF MS assays

OGT and OGA assays were carried out in 25 µL final total volume. For standard conditions: purified OGT or OGA (1 µM of each enzyme per reaction) was incubated with 25 µM of purified histone peptides or control peptide in PBS buffer (pH = 7.4) for 1 h at 37 °C; for reactions with OGT, UDP-GlcNAc (30 µM) was added. For high concentration conditions: purified OGT (5 µM) was incubated with 25 µM of purified histone peptides, full length histones or control peptide in PBS buffer (pH = 7.4) with UDP-GlcNAc (50 µM) for 1 h at 37 °C.

Histone methyltransferase assay was carried out as described: purified KMT enzymes G9a, GLP or SETD7 (2 µM of each enzyme per each reaction) were incubated with 100 µM of purified histone peptides in Tris–HCl (pH 8.0) and methyl donor SAM (500 µM for GLP/G9a and 200 µM for SETD7) in 30 µL total volume for 1 h at 37 °C.

Histone acetyltransferase assays were carried out by incubating histone peptides under standard conditions (2 µM GCN5 or PCAF, 100 µM peptide, 300 µM AcCoA) in the reaction buffer (50 mM HEPES, 0.1 mM EDTA, 1 mM DTT, pH = 8.0). The reactions were carried out in a final volume of 50 µL for 3 h at 37 °C.

All assays samples were quenched with MeOH (1:1), aliquoted and mixed 1:1 with α-Cyano-4-hydroxycinnamic acid (CHCCA) matrix dissolved in a mixture of H_2_O and ACN (1:1, v/v), and loaded onto an MTP 384 polished steel target to be analyzed by a UltrafleXtreme-II tandem mass spectrometer (Bruker). All samples were analyzed using reflector positive ion mode, while the full-length histone data were obtained using linear positive ion mode.

## Supplementary Information

Supplementary Information.

## Data Availability

The datasets used and/or analyzed during the current study available from the corresponding author on reasonable request.
