# Supplementary material for: Investigation of in vitro histone H3 glycosylation using H3 tail peptides
Source: Sci Rep. 2022 Nov 10;12:19251. doi: 10.1038/s41598-022-21883-0 (PMC9649660; doi:10.1038/s41598-022-21883-0)
Supplement: Supplementary file 1 — Supplementary Information. [file 41598_2022_21883_MOESM1_ESM.docx]

**Supporting Information**

**Investigation of *in vitro* histone H3 glycosylation using H3 tail peptides**

Jona Merx^1^, Jordi C. J. Hintzen^2^, Giordano Proietti^2^, Hidde Elferink^1^, Yali Wang^1,3^, Miriam R. B. Porzberg^2^, Daan Sondag^1^, Nurgül Bilgin^2^, Jin Park^4^, Jasmin Mecinović^2,^*, Thomas J. Boltje^1,^*

**Table of Contents**

| 1. | Experimental | S3 |
| --- | --- | --- |
| 2. | Synthetic schemes of the peptides | S4-S6 |
| 3. | Characterization of the peptides | S7-S10 |
| 4. | MALDI-TOF supplementary Figures | S11-S13 |
| 5. | SDS PAGE of OGT expression | S13 |
| 6. | Spectral data | S14-S17 |

**1. Experimental section**

**1.1 Methods**

^1^H and ^13^C NMR spectra for the building blocks and the intermediates were recorded on a Bruker AVANCE III (500 MHz ^1^H, 125 MHz ^13^C) equipped with a Bruker Prodigy BB cryoprobe in the solvent indicated at room temperature. Chemical shifts are reported in *δ* (ppm) units relative to the internal reference tetramethylsilane (Me_4_Si). For ^1^H NMR spectra, the following abbreviations are used to describe multiplicities: s (singlet), d (doublet), t (triplet), bs (broad singlet), dd (double doublet), and m (multiplet). Coupling constants are reported in Hertz (Hz) as a *J* value. NMR was carried out in 5 mm diameter Boroeco-5-7 tubes from Deutero (Kastellaun, Germany). NMR spectra were recorded at 298 K unless otherwise specified. Mass spectra were recorded on Thermo Finnigan LCQ Advantage Max. LC-MS was carried out on a Shimadzu LCMS-QP8000 (Duisburg, Germany) single quadrupole bench-top mass spectrometer operating in a positive ionization mode. The scanning range was m/z 50-2000. A gradient of MeCN/H_2_O containing 0.1 % formic acid was used. Samples were injected using a flow rate of 0.2 mL/min and eluted with 5-100 % in 50 min, infused in the Electrospray system. All compounds were routinely checked by TLC on Kieselgel 60 F254 (Merck, Darmstadt, Germany); spots were visualized under UV light (254 nm) and were stained Cerium Molybdate Stain or aqueous KMnO_4_ (depending on the reaction), followed by heating on a hot plate. Rf values were obtained with the indicated solvent mixtures. All solvents were reagent grade and, when necessary, were purified and dried by standard methods. The yields of the samples were calculated after drying the samples under high vacuum overnight and corrected for presence of residual solvents.

**1.2 Materials**

All commercially purchased reagents were used without further purification as delivered from the corresponding companies. Amino acids and the reagents were purchased from the following companies: Preloaded Wang resin (100-200 mesh) as the solid support and Fmoc-Lys(Boc)-OH were obtained from Novabiochem (Darmstad, Germany). Trifluoroacetic acid (TFA), Triisopropylsilane (TIS), 1-Hydroxybenzotriazole (HOBt), Tris-*d*_11_ solution, *S*-Adenosyl-L-Methionine (SAM), *α*-cyano-4-hydroxycinnamic acid, and *N,N′*-diisopropylethylamine (DIPEA), were purchased from Sigma Aldrich. Fmoc-allylglycine-OH, Fmoc-Thr(^t^Bu)-OH, and Fmoc-Gln(Trt)-OH were purchased from Carbosynth (Berkshire, UK). *N*,*N'*-Diisopropylcarbodiimide (DIPCDI) and piperidine were purchased from Biosolve chemicals (Valkenswaard, The Netherlands). Breipohl Resin [Fmoc-4-methoxy-4’-(-carboxypropyloxy)-benzhydrylamine linked to Alanyl-aminomethyl] (200-400 mesh) were purchased from Bachem AG (Bubendorf, Switzerland). Fmoc-Ala.OH.H_2_O and Fmoc-Asn(Trt)-OH were obtained from Iris Biotech (Marktredwitz, Germany). Fmoc-OSu was purchased from Chemicals Block (Santiago, USA). Fmoc-Ser(^t^Bu)-OH, Fmoc-Gly-OH, Fmoc-His(Trt)-OH, were purchased from Chem-Impex Int’l Inc (Illinois, USA). Fmoc-Arg(Pbf)-OH, and 1-[Bis(dimethylamino)methylene]-1H-1,2,3-triazolo[4,5-b]pyridinium 3-oxid hexafluorophosphate (HATU) were obtained from Fluorochem Ltd. (Derbyshire, UK). DMF and MeCN were purchased from Actu-All Chemicals b.v (Oss, The Netherlands).

**2. Synthetic schemes of the peptides**

**Supplementary figure S1.** Solid phase synthesis of the natural histone peptide H3K9. The same synthetic route was used to synthesize unnatural histone peptides that possess varieties at γ-site at position 2,4, 8, 9 and 14 of histone 3.

**Supplementary figure S2.** Histone peptides containing PTMs on position 2, 4, 8, 9 and 14 and combinations thereof.

**Supplementary figure S3.** Solid phase synthesis of the histone peptide H3 with S10GlcNac.

**Supplementary figure S4.** Solid phase synthesis of the control peptide.

**3. Characterization of the peptides**

**A B**


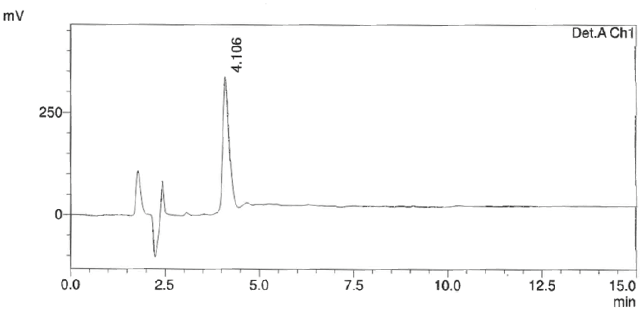

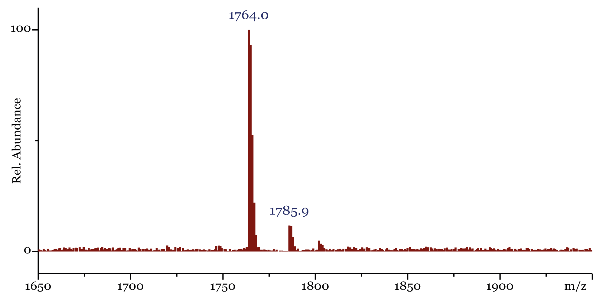


**C D**


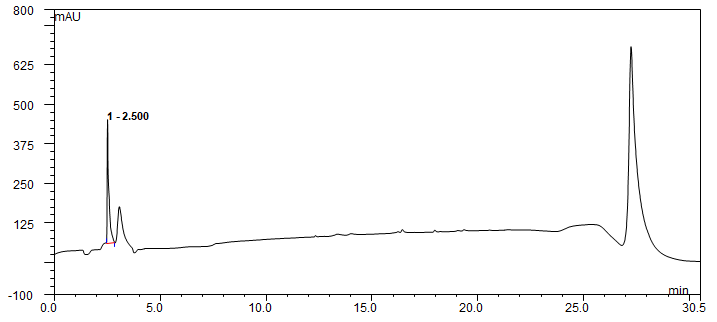

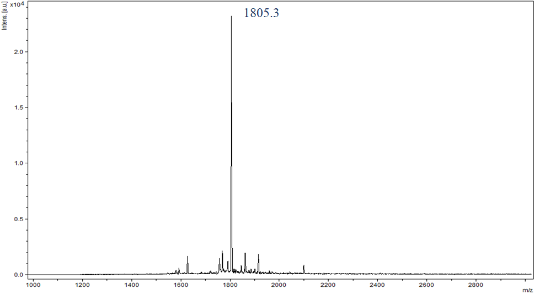


**E F**


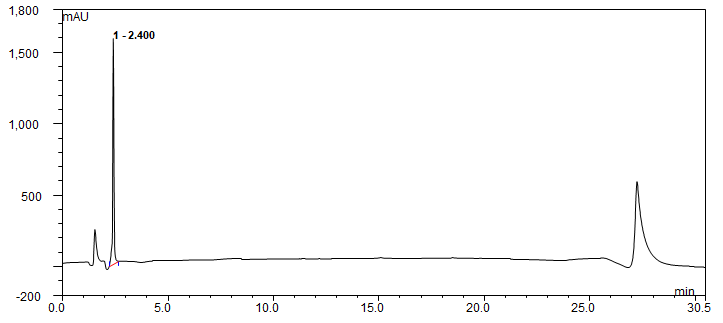

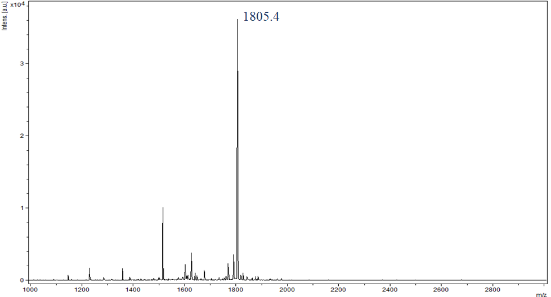


**G H**


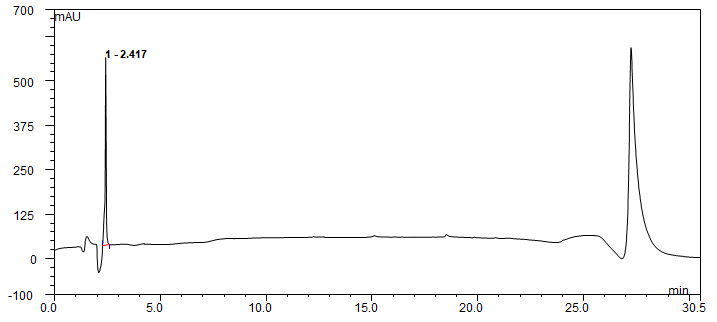

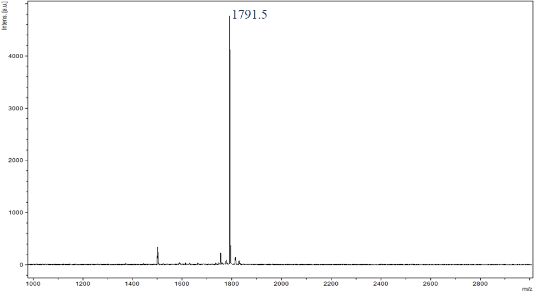


**Supplementary figure S5.** Characterization of glycosylated histone peptides after prep-HPLC purification. Analytical HPLC trace of A) H3S10GlcNAc and B) MALDI-TOF MS of H3S10GlcNAc, C) H3K9me_3_S10GlcNAc and D) MALDI-TOF MS of H3K9_me3_S10GlcNAc, E) H3K9_ac_S10GlcNAc and F) MALDI-TOF MS of H3K9_ac_S10GlcNAc, G) H3R8_me2a_S10GlcNAc and H) MALDI-TOF MS of H3R8_me2a_S10GlcNAc.

**A B**


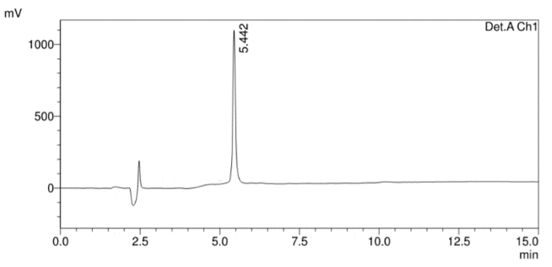


**C D**

**
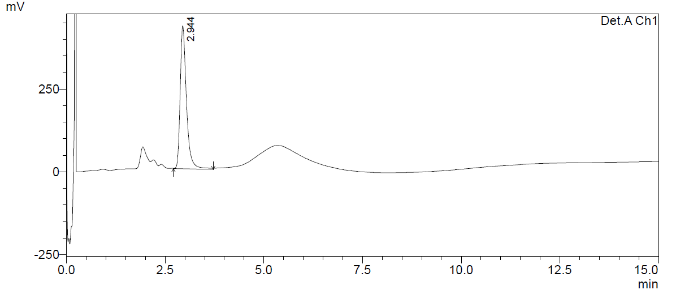
**

**Supplementary figure S6.** Characterization of histone peptides after prep-HPLC purification. Analytical HPLC trace of A) H3(1-15) and B) MALDI-TOF MS of H3(1-15), C) H3(23-37) and D) MALDI-TOF MS of H3(23-37).

**A B**

**
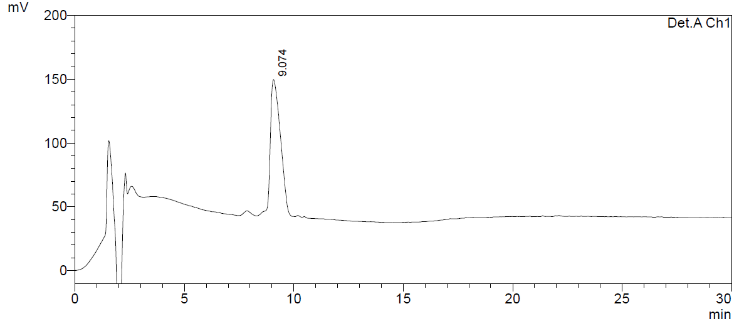
**

**C D**

**
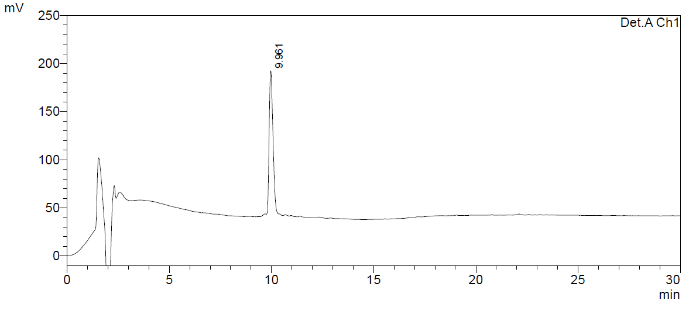
**

**Supplementary figure S7.** Characterization of Di- acetylated histone peptides after prep-HPLC purification. Analytical HPLC trace of A) H3K4_Ac_K14_AC_ and B) MALDI-TOF MS of H3K4_Ac_K14_AC_, C) H3K4_Ac_K9_AC_ and D) MALDI-TOF MS of H3K4_Ac_K9_AC_

**A B**

**
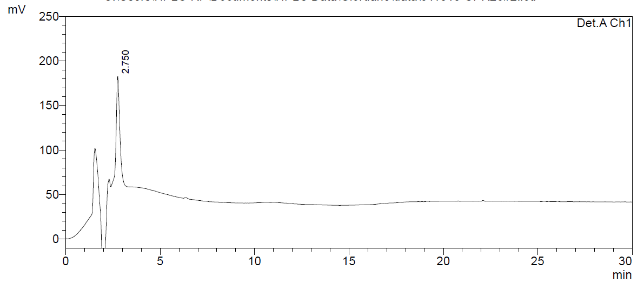
**

**C D**

**
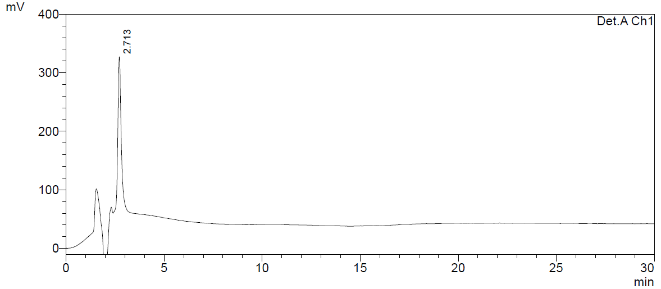
**

**Supplementary figure S8.** Characterization of acetylated histone peptides after prep-HPLC purification. Analytical HPLC trace of A) H3K9_AC_ and B) MALDI-TOF MS of H3K9_AC_, C) H3K14_Ac_ and D) MALDI-TOF MS of H3K14_Ac_

**A B**

_
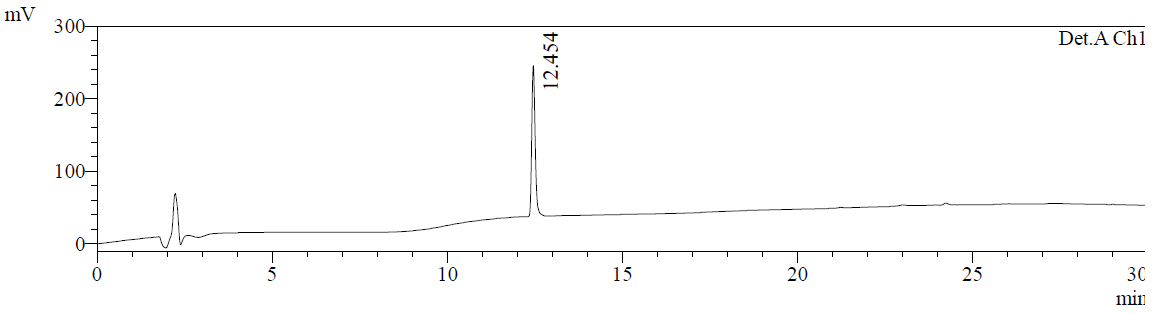
_

**Supplementary figure S9.** Characterization of control peptide after prep-HPLC purification. Analytical HPLC trace of A) CP and B) MALDI-TOF MS of CP.

**4. MALDI-TOF supplementary Figures**


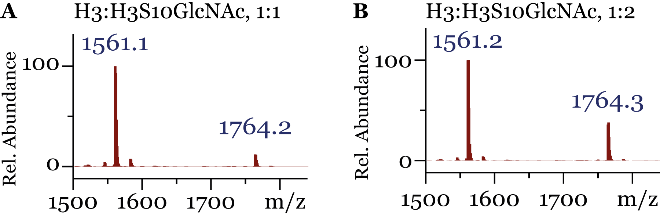


**Supplementary figure S10.** MALDI-TOF MS data showing relative abundance of **A**) H3 vs H3S10-GlcNAc (1:1) and **B**) H3 vs H3S10-GlcNAc (1:2).


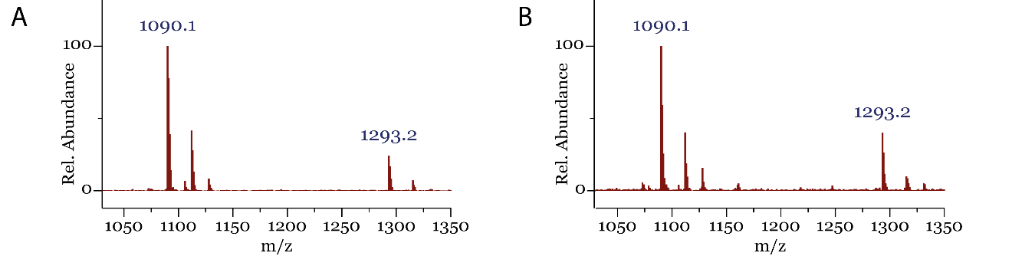


**Supplementary figure S11.** Control peptide after A) 1h incubation with OGT + UDP-GlcNAc and B) 3h incubation with OGT + UDP-GlcNAc.


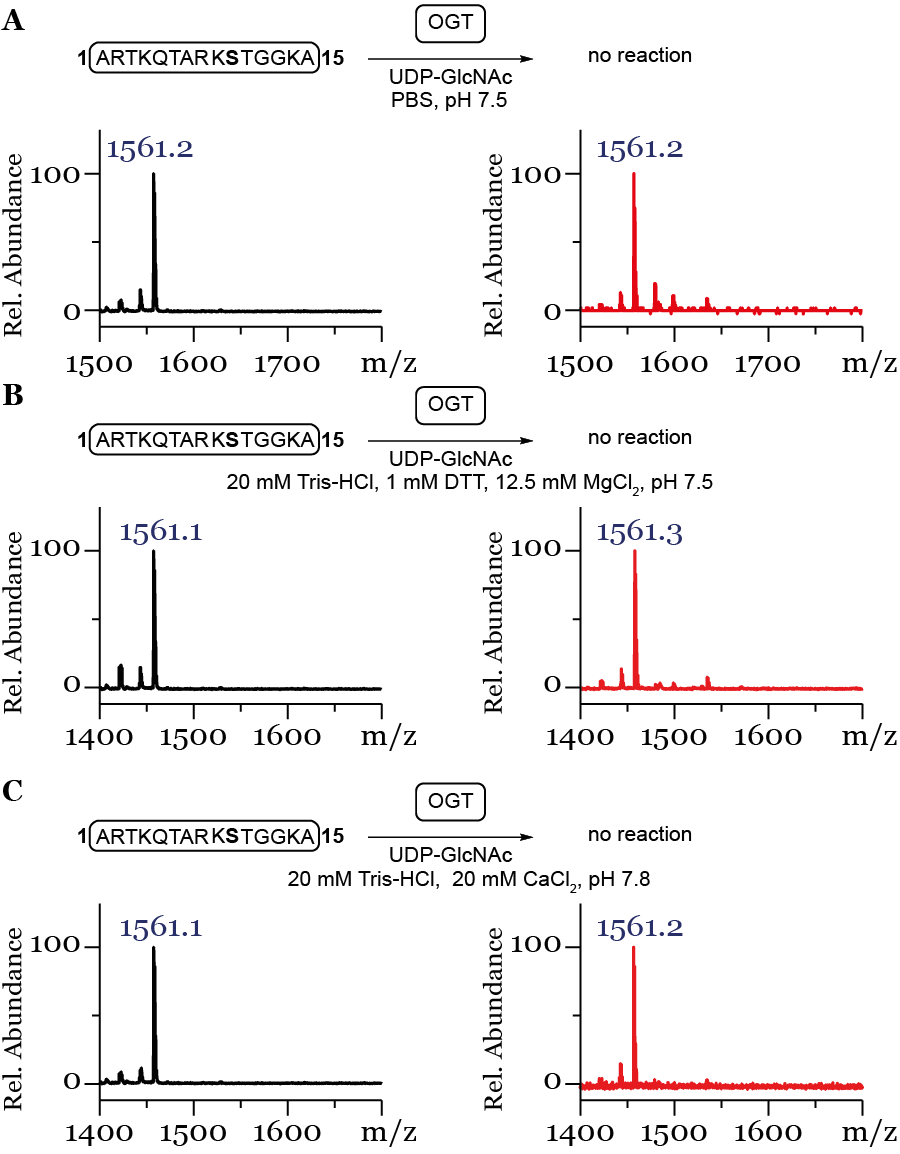


**Supplementary figure S12.** MALDI-TOF MS data showing no ability of OGT to catalyse histone H3 tail peptide in commonly used buffer for OGT catalysed *O*-GlcNAcylation. A) PBS buffer, pH 7.5, B) 20 mM Tris, 1 mM DTT, 12.5 mM MgCl_2_, pH 7.5 and C 20 mM Tris, 20 mM CaCl_2_, pH 7.8.

**
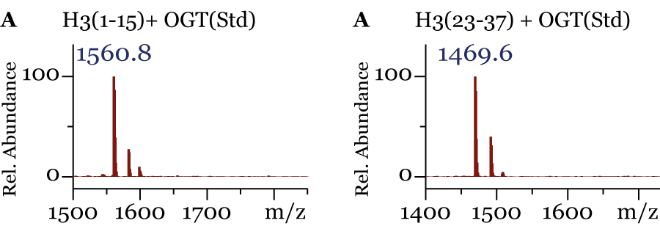
**

**Supplementary figure S13.** MALDI-TOF MS data showing peptides treated with OGT under standard conditions (Std) A) H3(1-15) + OGT(Std) B) H3(23-37) + OGT(Std).

**
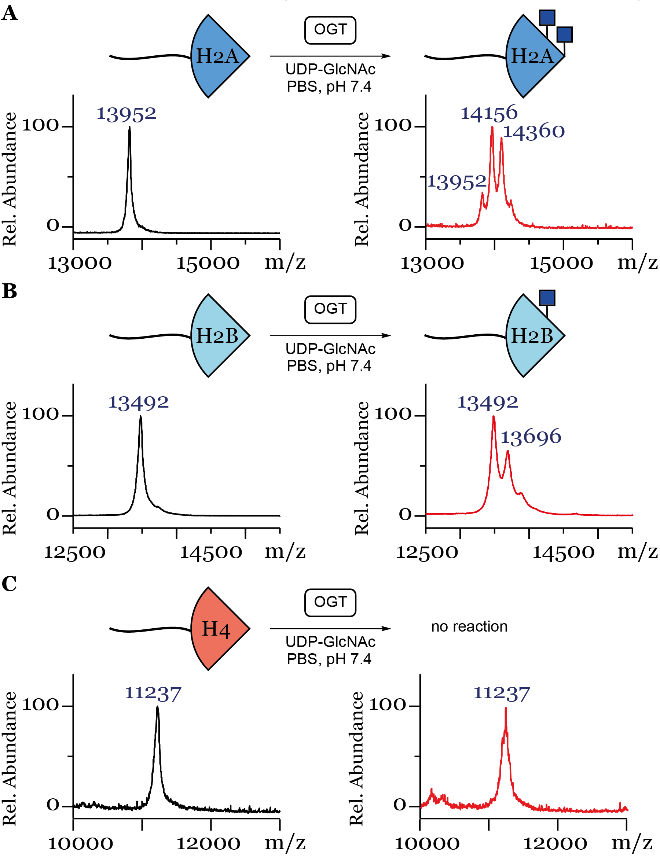
**

**Supplementary figure S14.** MALDI-TOF MS data showing full length histones A) H2A, B) H2B and C) H4 treated with OGT under optimal conditions (25 µM Histone, 5 µM OGT, 50 µM UDP-GlcNAc).

**5. SDS page gel of OGT expression**


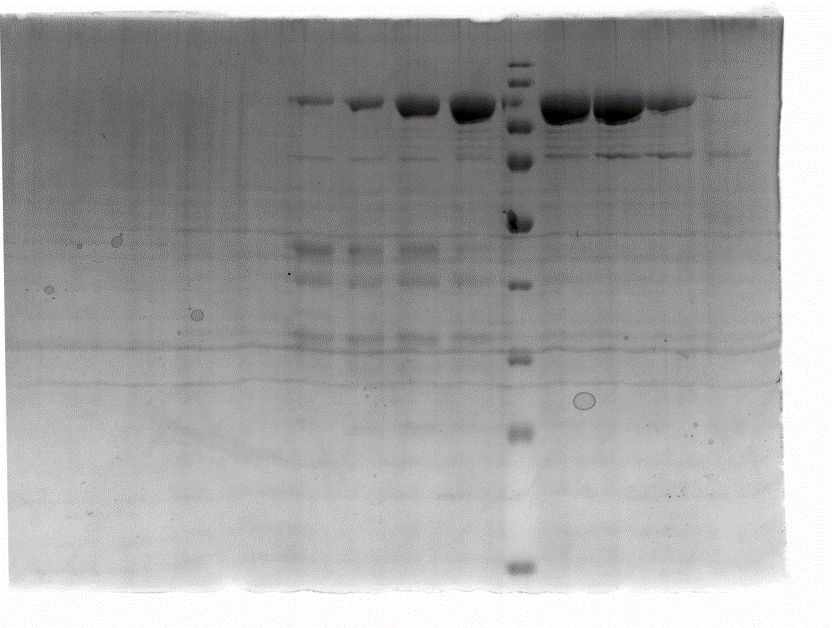


**Supplementary figure S15.** SDS Page of expressed OGT, the seven fractions were pooled and used as is. Ladder: pageruler prestained protein ladder 180 to 10 KDa.

**6. Spectral Data**
